# Research on Underwater Scene Reconstruction for Mobile Platforms Based on Rotating Scanning Sonar

**DOI:** 10.3390/s26092734

**Published:** 2026-04-28

**Authors:** Lei Tan, Lei Wang, Chaohe Chen

**Affiliations:** 1 School of Civil Engineering and Transportation, South China University of Technology, Guangzhou 510641, China; chenchaohe@scut.edu.cn; 2 Guangdong Institute of Intelligent Unmanned System, Guangzhou 511458, China; wanglei@gis.sia.cn

**Keywords:** unmanned surface vehicle (USV), rotating scanning sonar, three-dimensional reconstruction

## Abstract

High-precision underwater perception and scene reconstruction are critical techniques for marine surveying and resource exploration. Multi-sensor data fusion is currently the dominant method in underwater sensing. In this paper, a new approach for underwater sensing based on an integration of a 3D rotating scanning imaging sonar, an RTK (Real-Time Kinematic), and an IMU (Inertial Measurement Unit) systems onboard an unmanned surface vehicle (USV) is raised. By employing multi-sensor data fusion and image correlation calibration, combined with multi-view acoustic image synthesis, the system achieves accurate reconstruction of both water column and seabed scenes. The new system offers high reconstruction accuracy, and provides a cost-effective solution for scene reconstruction with a low requirement of the precise motion control of the USV platform. High-precision seabed imaging results have been validated through lake bed imaging tests.

## 1. Introduction

Systematic investigation of underwater environments holds significant scientific value for elucidating ecological characteristics, resource distribution patterns, and historical relics in oceans, lakes, and rivers. Marine intelligent unmanned systems—including remotely operated vehicles (ROV), autonomous underwater vehicles (AUV), and unmanned surface vessels (USV)—have become critical pieces of equipment for marine surveying, subaquatic search-and-rescue operations, and resource exploration. The autonomy, real-time operational capability, and safety of these systems rely on real-time high-precision environmental perception/reconstruction and autonomous obstacle avoidance [1,2]. Within optically constrained turbid waters, sonar systems serve as the primary technical means for achieving real-time high-precision environmental perception on marine unmanned platforms.

In turbid or dark underwater environments, only sound waves are capable of long-range, high-precision detection and imaging [3,4]. Therefore, many researchers both domestically and internationally have reconstructed the three-dimensional structure of underwater objects for applications based on sonar data to realize underwater target detection, localization, tracking, and navigation [5,6,7,8,9]. Jouvencel B et al. leveraged the high directivity of a single narrow-aperture sonar by integrating 54 “pencil-beam” sonar sensors into the same underwater vehicle to simulate 3D reconstruction [10]. Aykin et al. proposed a method for 3D reconstruction of underwater objects based on the synthesis of multi-view images from a 2D forward-looking sonar, which involves merging and intersecting point clouds from different viewpoints, and validated its effectiveness [11]. Chensheng et al. developed a 3D reconstruction method based on multi-beam forward-looking sonar data, converting the acquired sonar images into sparse point cloud format through threshold segmentation and range-constrained filtering, followed by Rao–Blackwellized particle filtering (RBPF) and grid map generation via AUV pose estimation [12]. Joe et al. proposed an autonomous underwater vehicle equipped with forward-looking sonar and profiling sonar, fusing data from both types to address the ill-posed problem in single-sonar 3D reconstruction, and validated its effectiveness in both tank and sea trials [13]. Daniel Filipe Campos et al. proposed the use of an autonomous surface vehicle integrating GPS, IMU, 3D LiDAR, and multibeam sonar to achieve simultaneous high-precision mapping above and below the water surface, with odometry errors of approximately 0.05–0.07 m and an environmental reconstruction cycle of 300 ms [14]. Tim Hansen et al. introduced a composite scanning method for mechanically scanned sonar, which embeds attitude information into single-beam data and uses online SLAM techniques to optimize the scanning process, thereby enabling real-time mapping [15]. Tim Hansen et al. also constructed a dataset for an unmanned underwater vehicle (UUV) integrating a mechanically scanned sonar (MSS), an inertial measurement unit (IMU), and a Doppler velocity log (DVL), with real-time localization achieved through a high-precision optical tracking system [16]. Zhang Lufeng et al. proposed a method for underwater pier inspection by combining 3D underwater sonar with multi-beam bathymetric sonar, achieving integrated 3D imaging of pier foundations and the surrounding riverbed through data fusion [17]. Huang Jiatao constructed a multi-source collaborative surveying framework integrating airborne LiDAR, UAV photogrammetry, and sonar bathymetry. By employing two key technologies—multi-source point cloud fusion for land areas and dynamic weight-based water depth fusion for shallow water—the framework effectively addresses issues such as land-water alignment offsets and insufficient data accuracy in shallow water [18]. Its effectiveness is validated in the Jiangmu Island experimental area. Christian Westerdahl proposed a GNSS-independent unified surveying system that integrates a LiDAR-inertial measurement unit and orthogonal dual forward-looking sonars on an autonomous surface vehicle, generating seamless three-dimensional maps from the seabed to the sky. Its real-time performance is validated in a real-world environment in Copenhagen [19]. Gabriele Vozza constructed a low-cost bathymetric data acquisition system by synchronizing sonar and GNSS via a smartphone, and established a machine learning-based three-dimensional modeling pipeline, which enables flexible and convenient reconstruction of natural and artificial underwater scenes with centimeter-level accuracy [20]. Collado-Gonzalez proposed a unified volumetric mapping framework that fuses stereo sonar and monocular cameras, eliminating elevation ambiguity through overlapping sonar fields of view and generating confidence-weighted 3D point clouds, thereby enabling reliable navigation and mapping for autonomous underwater vehicles under variable visibility conditions [21].

Active sonar systems exhibit significant directivity. To acquire full 3D spatial surveying data in underwater environments, two primary technical approaches are generally employed: mechanical scanning sonar and phased array sonar, each with distinct advantages and limitations. Two-dimensional or 3D phased array sonar imaging offers high imaging efficiency. However, it is hampered by complex and costly equipment, limited interpretability of dynamic imagery, and the difficulty of performing real-time spatial integration. Conversely, mechanical scanning sonar features a simple structure and cost efficiency. The low speed of sound in water necessitates dynamic scene scanning during imaging. Acquiring 3D spatial information is time-consuming, leading to a low frame refresh rate and high requirements for the attitude and motion control accuracy of underwater carrier platforms, resulting in severe limitations on its application due to environmental and scenario constraints. Consequently, a universally effective underwater sensing system or solution remains lacking in the geomatics area.

To address the aforementioned challenges and further enhance the real-time performance and accuracy of underwater 3D reconstruction, this paper proposes an underwater detection and reconstruction system based on an unmanned surface vehicle (USV) platform. The system employs a self-developed rotating scanning sonar, which utilizes phased array beamforming technology to improve scanning speed, and achieves wide-angle 3D spatial imaging within a single scan cycle by leveraging multi-beam and vertical resolution capabilities. An underwater surveying system integrating RTK, IMU positioning, and motion sensing was constructed, and data collection was conducted in a small lake in Guangzhou. High-resolution images of parts of the lakebed were generated through post-processing methods, validating the effectiveness of the proposed system and methodology.

## 2. Implementation of the USV Surveying System

### 2.1. USV Surveying System

**Figure 1:**
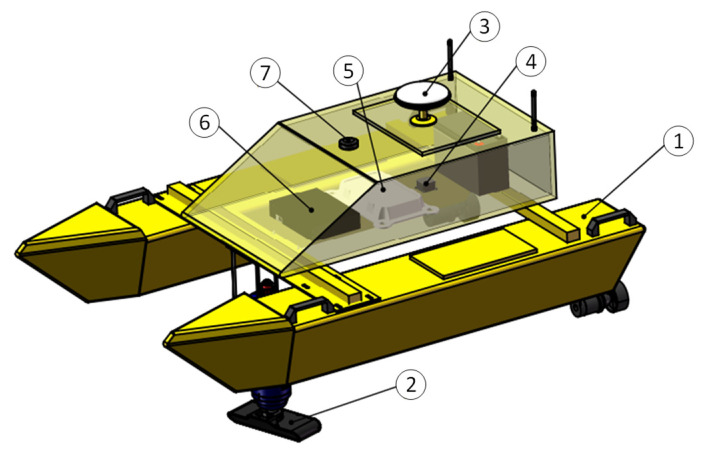
Structural Composition of the Dynamic Underwater Surveying System.

To improve the real-time capability and accuracy of underwater environmental reconstruction, a USV-based surveying system is developed for technical validation. As shown in Figure 1, the USV system consists of the following components:

(1) Surface Dynamic Measurement Platform ①: An unmanned surface vehicle serves as the mobile carrier for the sensing equipment.
(2) Rotating Scanning Imaging Device ②: A mechanically scanned 3D imaging sonar, fixed beneath the hull of the USV. The sonar probe rotates 360° around the vertical axis for scanning, forming a circular sector-shaped detection area.
(3) Positioning and Attitude Determination Module: This includes a high-precision RTK receiver ③ and an IMU inertial measurement unit ④, providing centimeter-level spatial positioning, heading, elevation, and real-time hull roll, pitch, and yaw angle data.
(4) Data Fusion Processing Module ⑤: Installed onboard, this module is responsible for receiving data from all sensors and executing core processing algorithms.
(5) Communication, Power Supply, and Auxiliary Module: This includes a data storage module ⑥, a wireless transmission module ⑦, and a shore-based display and control module. Module ⑥ is used to store raw data, module ⑦ wirelessly transmits processed results to module for real-time 3D image display.

### 2.2. Underwater Reconstruction Coordinate Systems

In underwater 3D reconstruction, an accurate description of the coordinate system is indispensable. It provides a unified mathematical framework and spatial reference for multi-source sensor data such as sonar and IMU, serving as the prerequisite for precise data fusion and the generation of seamless and metrically accurate 3D models.

(1) Sonar Coordinate System

To better achieve coordinate transformation of underwater environmental point cloud data, the sonar coordinate system is defined with the center of the sonar transducer probe as the origin. The *X*-axis aligns with the heading direction of the unmanned surface vehicle (USV), the *Y*-axis points to the right side of the sonar, and the *Z*-axis is perpendicular to the sonar’s track and points downward.

(2) USV Coordinate System

Since sensors such as the sonar, RTK, and IMU are fixed on the USV platform, their respective reference coordinates vary depending on their mounting positions. Therefore, a unified USV coordinate system is typically established for multi-sensor data fusion. This system takes the geometric center of the USV as the origin, with the heading direction as the *x*-axis. The roll angle φ rotates around the *x*-axis, with a downward tilt of the starboard side defined as positive. The *y*-axis points to the starboard side of the USV, and the pitch angle θ rotates around the *y*-axis, with bow-up defined as positive. The *z*-axis points toward the lakebed, perpendicular to the oxy plane in the USV coordinate system, and the heading angle ψ rotates around the *z*-axis, with clockwise defined as positive.

(3) Planar Projection Coordinate System

In this study, the initial departure point of the USV serves as the origin of the planar projection coordinate system. The N-axis points to true north, the E-axis points to true east, and the D-axis is perpendicular to the coordinate plane formed by the N- and E-axes.

**Figure 2:**
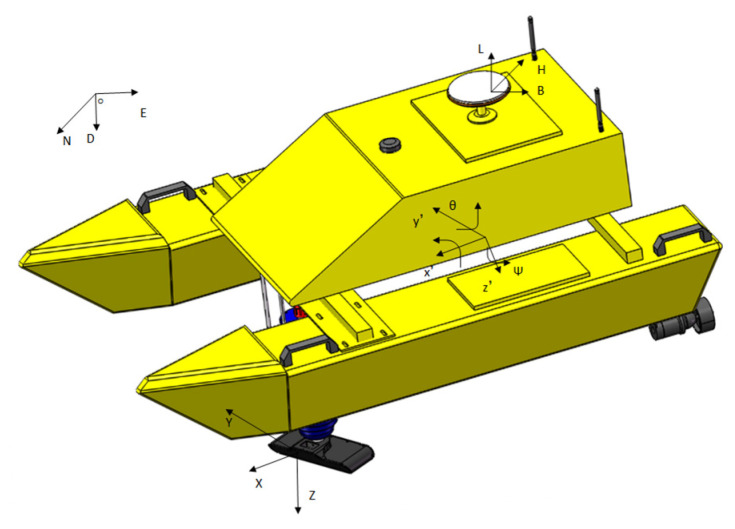
Schematic Diagram of Coordinate Systems.

As the sonar is the core device of the USV underwater detection system, its data volume is several orders of magnitude higher than that of other sensors. To reduce the computational complexity of spatial coordinate transformation for imaging, the USV platform coordinate system is omitted here. Instead, only the sonar coordinate system is used, with RTK and IMU data from the same timestamp transformed into the sonar coordinate system. The relevant coordinate relationships are illustrated in Figure 2.

Among these, the geodetic coordinates obtained by the RTK device are (B, L, H), where B represents latitude, L represents longitude, and H denotes ellipsoidal height. The sonar coordinate system is denoted as osxsyszs, while φ, θ, and ψ represent the roll, pitch, and yaw angles of the vessel coordinate system (*x*’, *y*’, *z*’), respectively.

A local horizontal coordinate system is established with the origin at the sonar coordinate system. R is the phase-center of the RTK, denoted as P_RTK_, has a position (*xR0*, *yR0*, *zR0*) in this local ENU (East-North-Up) coordinate system. Since the IMU remains stationary relative to both the sonar and the vessel, the Yaw-Pitch-Roll angles provided by the IMU correspond to rotations around its own *X*-axis, *Y*-axis, and *Z*-axis, which are also represented as *φ*, *θ*, and *ψ*, respectively.

In this study, the phase center of the RTK is referenced to a Cartesian coordinate system with the Earth’s center as the origin. The latitude, longitude, and height obtained by the RTK for a given point in the geodetic coordinate system can thus be converted into the ENU coordinate system and expressed as (*E*, *N*, *U*). The rotation matrix of the IMU in the ENU coordinate system, denoted as $R_{ENU}^{IMU}$, is obtained by multiplying the three Euler angle rotation matrices. The translation vector from the RTK to the sonar origin is denoted as $t_{SON}^{RTK}$, and the sonar point cloud coordinate $P_{son}$ in the local ENU coordinate system can be expressed as $R_{ENU}^{SON}$.

(1)  = SONPRTK+RENUIMU·RENUSON

By utilizing the inverse conversion algorithm from ENU (East-North-Up) to WGS84 in conjunction with the above equation, point cloud data can be rapidly transformed from a dynamic coordinate system to a static North-East-Up coordinate system. This enables the generation of underwater environmental point clouds with georeferenced coordinates, establishing a unified spatial reference for 3D reconstruction.

### 2.3. Sonar Model

**Figure 3:**
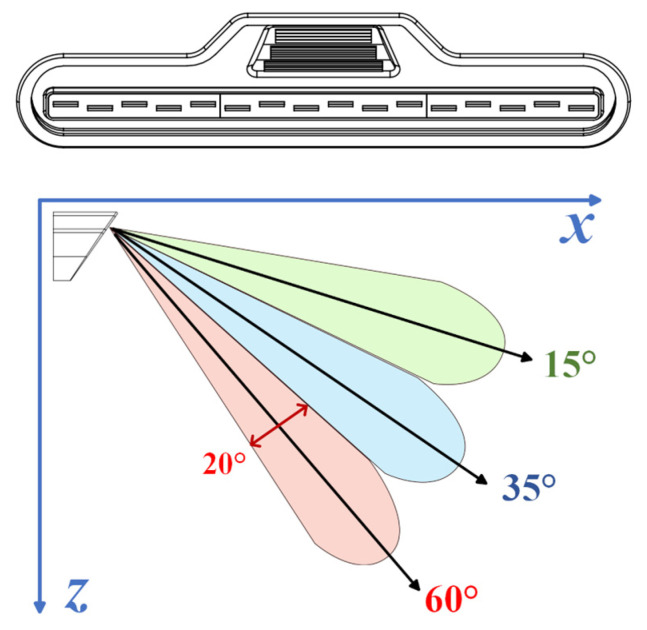
Schematic diagram of the XYT3000 sonar array configuration and vertical beam pattern. The color green, blue and red refers to 3 different beams generated by the transducers.

The sonar model employs the independently developed 3D scanning sonar XYT3000 from ZynSonic Ltd. (Guangzhou, Guangdong Province, China), as shown in Figure 3. This sonar system utilizes three sets of transmitting transducers with different vertical orientations, whose beam centers are directed at 15°, 35°, and 60°, respectively, forming a vertical profile of approximately 65°. The sonar receiving array is a 16-element dual linear array, which achieves fine horizontal resolution via beamforming, while the phase difference of echoes from the dual arrays is used to estimate vertical orientation. The acoustic array is driven by a stepper motor to rotate around the central axis of the device housing. The transmitting array emits short pulses at preset intervals, and the receiving array captures the echo signals. After synthesizing multiple PINGs, the system provides 3D imaging results of underwater targets.

Unlike many popular and traditional sonar systems, XYT3000 possesses true 3D imaging capability, which fundamentally distinguishes it from mechanically scanned sonar (MSS) and forward-looking sonar. Compared with the BV5000, the use of a blazed array in the XYT3000 system imposes constraints on dynamic platform performance, which is a well acknowledged limitation. Due to its high resolution, the system is not limited to topographic mapping; it enables comprehensive scene perception including sediment types, seafloor features, underwater structures, and fish schools.

Most MSSs and forward-looking sonar systems typically focus on either bathymetry or obstacle detection. In contrast, XYT3000 provides high-resolution 3D imaging that captures both static structures and moving targets (e.g., fish) simultaneously.

The specifications of the XYT3000 sonar are shown in Table 1.

The image space of the sonar is established within the sonar coordinate system. After the acoustic wave data undergoes IQ quadrature demodulation and down-sampling filtering, the upper and lower linear arrays perform beamforming, respectively, to produce $b_{up}(t,\theta_{m,k})$ and $b_{down}(t,\theta_{m,k})$:

(2) $$\left\{\begin{array}{c} b_{up}\left(t,\theta_{m,k}\right)=\sum_{n=1}^{N}x_{2n-1}\left[t-\frac{2n-1}{c}\cdot d\cdot \sin\left(\theta_{m,k}-k\delta \right)\right]\\ b_{down}\left(t,\theta_{m,k}\right)=\;\sum_{n=1}^{N}x_{2n}\left[t-\frac{2n}{c}\cdot d\cdot \sin(\theta_{m,k}-k\delta )\right] \end{array}\right.$$

where: n=1,...,2N., $x_{n}(t)$ is the receive time domain signal of the nth array elements, d is the element spacing, **c** is the sound speed in water.

(3) $$\theta_{m,k}=m\varepsilon -\frac{1}{2}M+k\delta ,\;m=1,\ldots ,M.$$

$\theta_{m,k}$ denotes the horizontal angle in the sonar coordinate system, with ***m*** as the beam index, ***k*** as the PING number, ε as the angular spacing between beams, and δ as the rotation angle per Ping. For simplicity in signal processing, it is common to set:

(4) δ=M⋅ε

The intensity image can be obtained by summing the outputs of the upper and lower arrays:

(5) $$b\left(t,\theta_{m,k}\right)=b_{up}\left(t,\theta_{m,k}\right)+b_{down}(t,\theta_{m,k})$$

The phase difference between corresponding outputs of the upper and lower arrays is calculated as:

(6) $$\eta \left(t,\theta_{m,k}\right)=arg\left(b_{up}\left(t,\theta_{m,k}\right)\cdot b_{down}^{*}\left(t,\theta_{m,k}\right)\right)$$

Within each transmitted beam, the phase difference corresponds to a finely resolved vertical angle:

(7) $$\phi \left(t,\theta_{m,k}\right)=\arcsin\left(\frac{1}{2\pi h}\eta \left(t,\theta_{m,k}\right)\cdot \lambda_{i}\right)$$

where $\lambda_{i},i=1,2,3$; represents the wavelength corresponding to each beam, and ***h*** is the vertical spacing between the dual linear arrays. Given that phase is susceptible to noise interference, spatial filtering of the point cloud image is necessary.

By combining the intensity and phase images from the three transmitted beams covering the spatial domain and converting the measurements to the range dimension, a spatial point cloud image in the sonar’s own coordinate system can be obtained: $b(r,\theta_{m,k},\varphi )$. Where ***φ*** is the vertical angle of the spherical coordinates.

Considering that the position and attitude of the sonar may change during measurement, which is equivalent to the extension and overlapping of the spatial point cloud image in the geodetic coordinate system, correction based on auxiliary sensor information is required.

## 3. Sonar Imaging Space Under Platform Motion Conditions

When the platform moves or sways, the imaging area of the scanning sonar also changes accordingly. The specific imaging area is determined by the sonar/platform attitude, platform motion speed, distance from the seabed (altitude), as well as the seabed slope or the spatial structure of the underwater scene. Although 3D scanning sonar does not require the platform to move in a straight line and is particularly suitable for stationary observation or slow movement toward a target, for simplicity in analysis, the following explanation still assumes the platform is in a straight-line navigation state to illustrate the relationship between the imaging area of the 3D scanning sonar and motion parameters.

Assume an unmanned surface vehicle (USV) is equipped with a 3D scanning sonar and moves along a straight line at a speed V. Simultaneously, the seabed is assumed to be a flat plane, with R representing the slanted range of the sonar within its imaging range. The scanning line equation:

(8) $$\left\{\begin{array}{c} x=R\cos\left(\theta_{v}t\right)+vt,\\ y=R\sin\left(\theta_{v}t\right); \end{array}\right.$$

**Figure 4:**
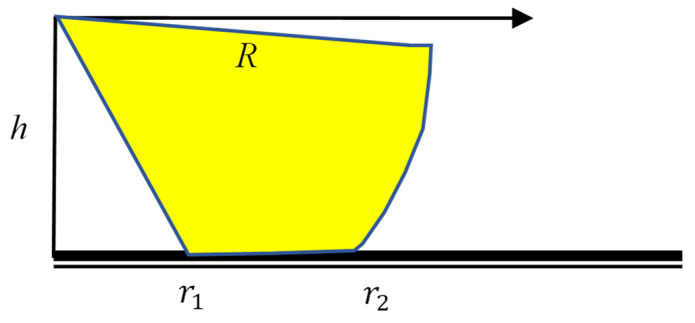
Water Body Coverage Area on the Vertical Plane shown in color yellow.

The $\theta_{v}$ is the rotation speed of the sonar probe. Therefore, the coverage area of the sonar over the water volume is shown in Figure 4.

Where $r_{1}$ and $r_{2}$ can be derived as:

(9) $$\left\{\begin{array}{c} r_{1}=h\;cot(\phi_{e}),\\ r_{2}=\sqrt{R^{2}-h^{2}}; \end{array}\right.$$

**Figure 5:**
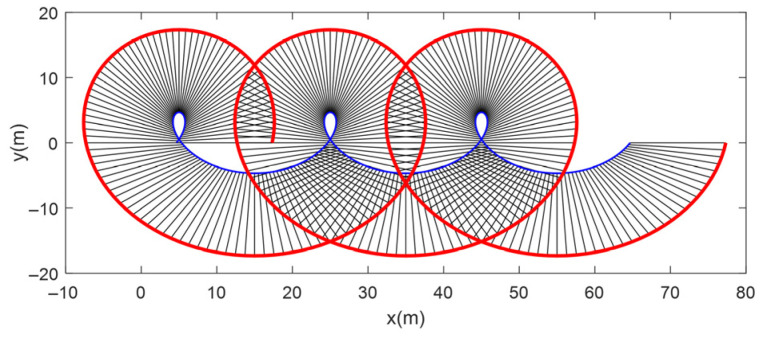
Seabed Coverage Map During Platform Motion (Counterclockwise Scanning Condition).

Assuming the sonar is positioned 10 m above the seabed with a detection range R = 20 m, a vertical downward viewing angle of $\varphi_{e}=65^{{}^{\circ}}$, $\theta_{v}=2\pi /10$, and a platform speed ***v*** of 4 kn. The seabed scanning coverage area can be derived as follows:

Overlapping scanning areas exist on the starboard side along the direction of travel. As referenced in Figure 5, the scanning zones indicated by the red and blue lines cover the water volume. A blind zone exists on the port side. When the platform’s speed decreases, the overlapping area of the red lines expands until the blind zone is eliminated. Therefore, if the observation target is located on the port side, the rotation direction should be switched to clockwise.

### 3.1. Impact of Moving Platform Sway

When the mobile platform is an unmanned surface vehicle (USV), it is subjected to irregular rolling and pitching due to wave effects on the sea surface. In terms of amplitude, rolling is generally more significant than pitching. For simplicity, simulations are conducted here under rolling conditions of 5°and 10°, respectively:

(10) $$\left\{\begin{array}{c} r_{1}=h\;cot(\phi_{e}+\phi_{roll}(t)),\\ r_{2}=\sqrt{R^{2}-h^{2}}; \end{array}\right.$$

**Figure 6:**
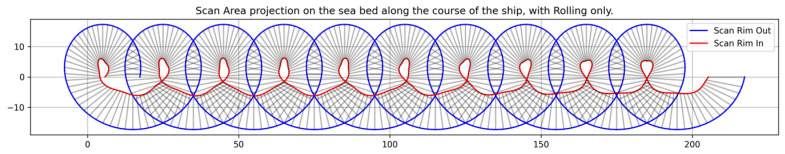
Sway diagram of the moving platform.

Assuming the rolling $\varphi_{roll}(t)$ follows a periodic sinusoidal function, the sway diagram is shown in Figure 6.

### 3.2. Seabed Slope and Rolling Scenarios

**Figure 7:**
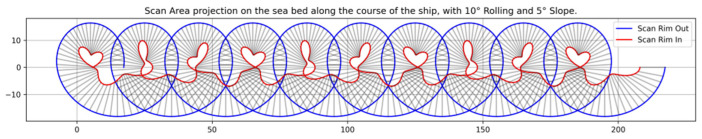
Diagram of Moving Platform Sway.

Figure 7 shows the seabed scanning results under the simultaneous conditions of an inclined seabed and platform sway, with simulation settings consistent with Section 2.3. The results are presented directly here.

### 3.3. Data Processing Methods

**Figure 8:**
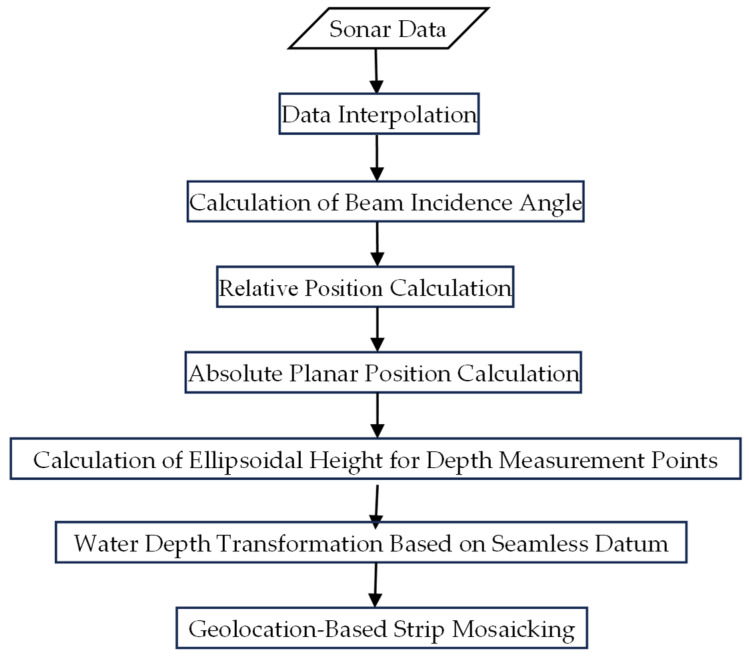
Data Processing Workflow for the Mobile Platform.

The data processing procedure can be referred to in the flowchart in Figure 8. First, interpolate the positioning and attitude data according to the transmission and reception time of each beam. Then, based on the attitude data at the moments of beam transmission and reception, calculate the spatial incidence angle of the beam. Next, determine the position of the beam point relative to the acoustic center of the sonar using the spatial incidence angle and the two-way travel time of the echo. Subsequently, compute the absolute position of the beam point in the geographic coordinate system by combining the absolute position of the sonar’s acoustic center at the transmission moment and the relative position of the beam point. Finally, calculate the ellipsoidal height of the depth measurement point based on the ellipsoidal height of the sonar’s acoustic center and the depth of the echo point relative to the sonar, and derive the absolute water depth of the echo point using a seamless datum.

## 4. Experiments and Data Analysis

### 4.1. Sonar Technical Parameters and Index Evaluation Test

**Figure 9:**
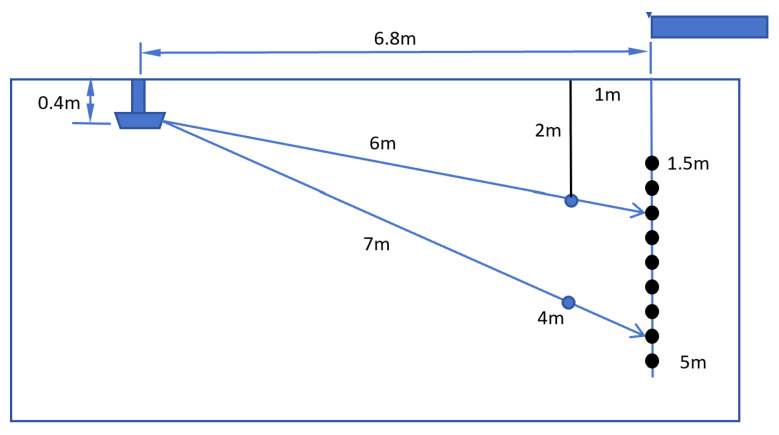
Schematic Diagram of Index Verification Test. The solid blue inverted T-shape on the left indicates the sonar probe, and the solid blue rectangle on the right indicates the test platform. A vertical line hangs down from the test platform, with several metal balls suspended.

The XYT3000 supports three operating transmit frequencies: 900 kHz, 1000 kHz, and 1100 kHz. It employs a composite waveform consisting of CW and Chirp signals, with the Chirp signal having a bandwidth of 50 kHz. The vertical beamwidth of the transmit beam for each frequency band is 20°, and the horizontal beamwidth is 1.125°. After signal processing, the target resolution is 2° in the vertical direction and 0.5° in the horizontal direction, with a range resolution of 1 cm and a maximum operating range of 40 m. The schematic diagram of the index verification test in the anechoic tank is shown in Figure 9.

The sonar probe is deployed at a depth of 0.4 m. The test target is a steel ball with a diameter of 3 cm, placed at a depth of approximately 4 m. The calculated slant range from the steel ball to the sonar is close to 7 m.

**Figure 10:**
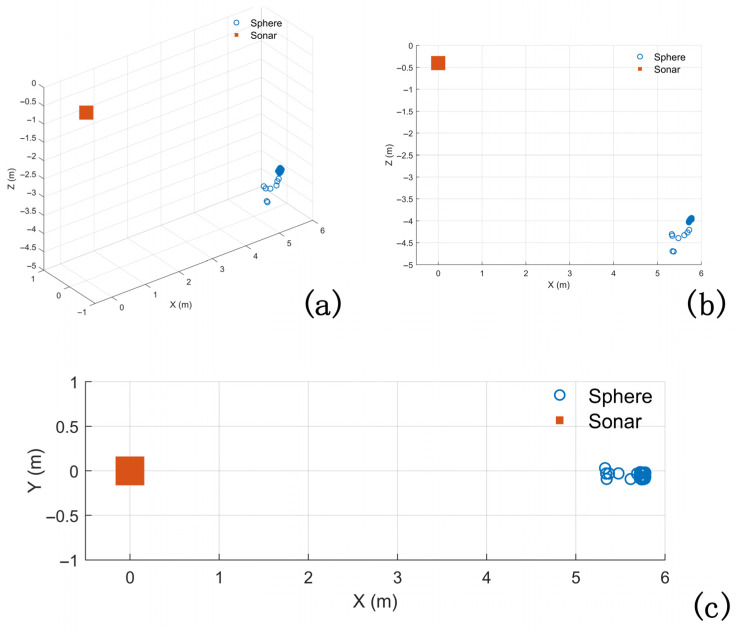
Steel Sphere Position Estimating Results. (**a**) 3D Perspective, (**b**) Side Perspective, (**c**) Top Perspective of the relative position of the sonar and the sphere.

A total of 80 frames of measurement data were collected, and the estimated results of the steel ball target are plotted in Figure 10. It can be seen that the estimation error in the horizontal bearing is very small. The depth estimation results are mostly around 4 m, and the horizontal distance is approximately between 5.5 m and 5.8 m, which is consistent with the actual deployment. The deviation in the steel ball position may be caused by the swing of the rope used to fix the steel ball.

For further quantitative analysis of the steel ball estimation results, 20 groups of data with stable horizontal bearing estimates were selected (to ensure the steel ball is practically stationary), as shown in Table 2. It can be seen that the horizontal bearing estimation error is less than 0.01°, the vertical bearing estimation error is approximately 0.13°, and the slant range estimate is stable at 6.762 m. The above results demonstrate that the XYT3000 sonar features excellent underwater resolution. Based on the high-resolution panoramic scanning sonar, it can provide accurate data support for subsequent underwater scene reconstruction.

### 4.2. Experiments

**Figure 11:**
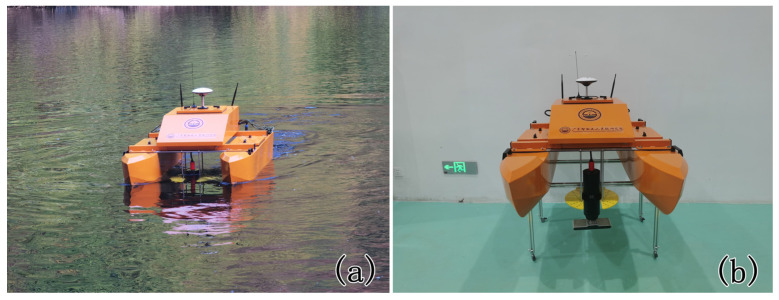
Dynamic Underwater Surveying System. (**a**) Photograph of Lake test. (**b**) Photograph of Actual assembly.

To validate the feasibility and effectiveness of the above-mentioned solution for underwater 3D reconstruction of the seabed and structures, experiments were conducted at a lake near Mingliyuan Farm in Nansha, Guangzhou. This lake is relatively large in scale and has considerable depth. Following the aforementioned scheme, an unmanned surface vehicle (USV) surveying system was constructed, as shown in Figure 11. Figure 11a depicts the actual assembly photograph of the underwater mapping system, and Figure 11b shows the lake test photograph.

**Figure 12:**
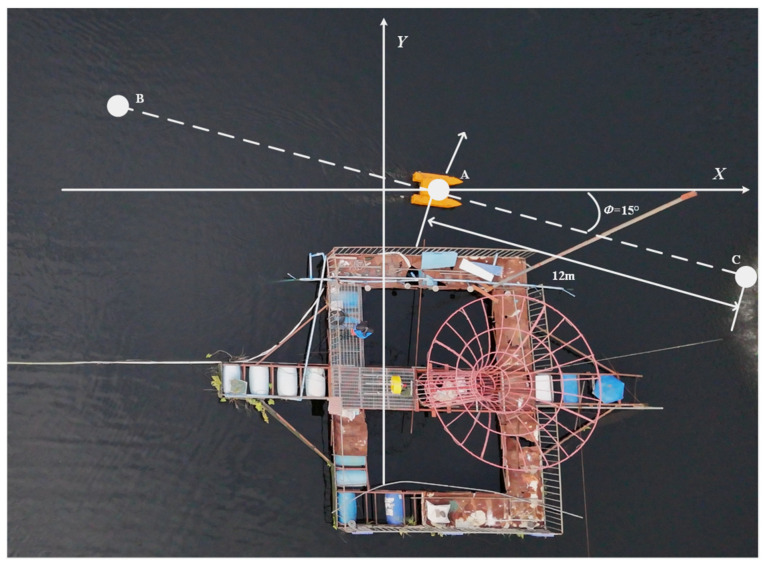
Schematic diagram of the test points.

In the experiment, the XYT3000 sonar was installed symmetrically relative to the center of the unmanned surface vehicle (USV), with no angular deviation in its attitude. The USV was also equipped with a positioning system, attitude sensors, and other devices. The GNSS/RTK Receiver features specifications shown in Table 3, and the positioning data frequency was 20 Hz, while the attitude data frequency was 1 kHz. The time synchronization error between different devices was less than 10 ms. After moving to the designated position (as shown in Figure 12), the USV remained stationary, and the sonar was activated to scan the underwater portion of the lake. During the test, the scanning range at each test position covered a diameter of 24 m. Then the acquired sonar scanning data were saved in bin files for subsequent use. Finally, to achieve rapid fusion of intensity and phase images, the paper adopts a direct concatenation method. The operational steps are as follows: register and normalize the images, directly concatenate the images along the channel dimension, and finally generate point clouds based on the images.

### 4.3. Experimental Data Analysis

**Figure 13:**
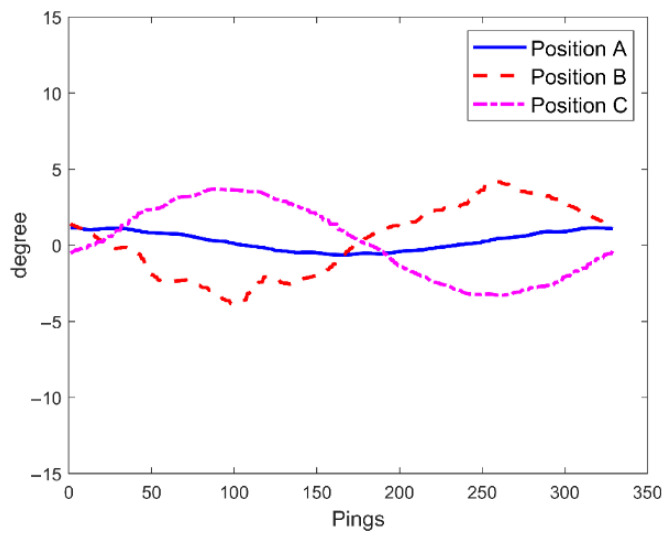
IMU Tilt Angle Diagram for Three Scanning Cycles.

Figure 13 shows the measurement results from the system at the floating platform location at different times. It can be observed that the main underwater terrain features in both scanning results are generally consistent. The depth near the origin is approximately 7 m. Points with XY coordinates (0, −5), (−5, −8), and (−15, −12) were selected, and the calculated depth difference between the two scans is 0.085 m. Variations in the shallower point clouds are mainly caused by fish movement in the lake (resulting in changes in sonar echo intensity).

**Figure 14:**
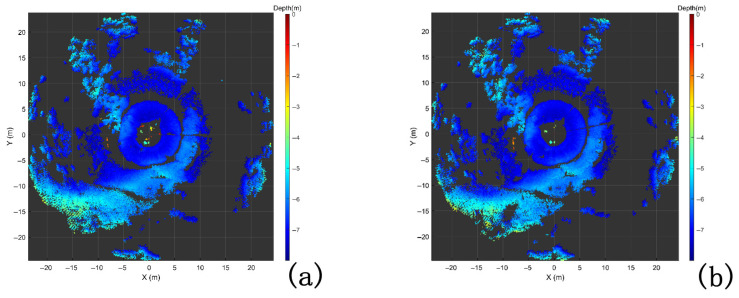
Scanning Results at Test Point A. (**a**) Data acquired at local time 15:31:55. (**b**) Data acquired at local time 15:32:12.

Blind zones in the results are due to terrain blocking the acoustic waves. Figure 14a and Figure 14b, respectively, present the subaqueous scanning data acquired at surveying point A at local time 15:31:55 and 15:32:12. By conducting comparative analysis of two adjacent datasets collected at identical geographical coordinates but distinct temporal points, the measurement consistency of the surveying system/methodology is examined.

**Figure 15:**
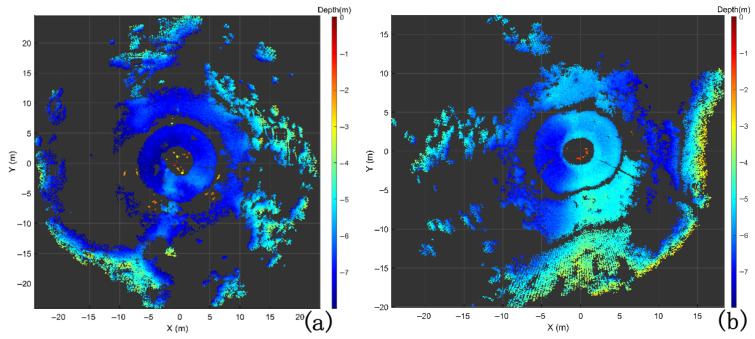
Underwater scanning results at different measurement points. (**a**) Measurement point B, (**b**) Measurement point C. The color bar shows the depth of the scanned targets.

For underwater topographic mapping at different locations, in addition to measurement point A, measurement points located 12 m to the left and right of point A were selected as multi-point test sites based on point A, and data fusion was performed. To ensure the accuracy of data fusion, the geodetic coordinates of the above three measurement points were calculated using the positioning module of the unmanned surface vehicle. The underwater scanning results of the system at measurement points B and C are shown in Figure 15. It can be observed that by moving the system position, the blind-zone information in the previous measurement results at the floating platform location, such as the blank area near the coordinate origin (directly below the sonar), could be supplemented.

To verify the reliability of the system’s depth measurements, the depth results at the same coordinate point obtained from different measurement locations were compared, as shown in Table 4. It can be observed that for the coordinate point (−0.85, −2.65), the depths calculated from the three measurement points are 6.76 m, 6.64 m, and 6.51 m, respectively, with a maximum difference of 0.25 m. The results are generally stable and reliable.

A direct concatenation strategy is adopted. The detailed steps are as follows: First, spatial registration and grayscale normalization are performed on the intensity and phase images. Secondly, the two images are directly concatenated along the channel dimension to generate a two-channel fused image; finally, a 3D point cloud is generated based on the fused image. This method is simple to implement, computationally efficient, and suitable for real-time applications.

**Figure 16:**
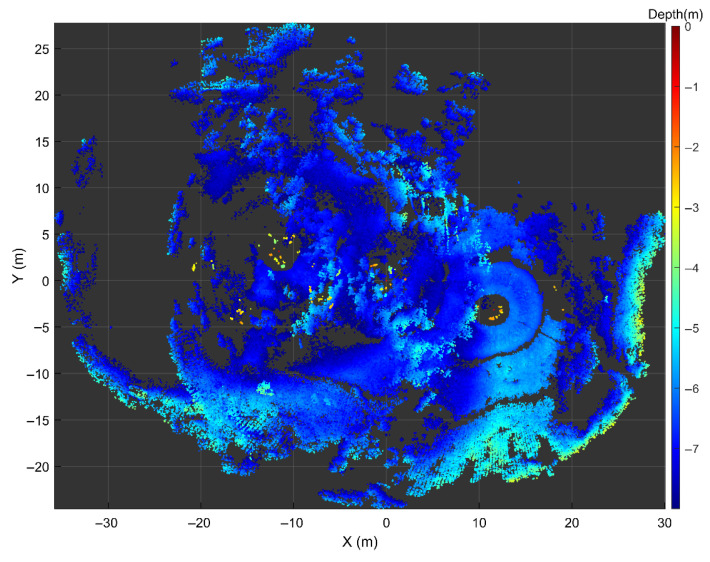
Stitched scanning results from three survey points.

After merging the scanning data from the three survey points, Figure 16 shows the stitched result of the data from these points. Due to the system’s detection range of 24 m, there is a certain overlapping area among the data from each survey point. For the region spanning from −20 m to 20 m, multi-point surveying can achieve coverage of at least 80% of the underwater area. Peripheral blind zones can be supplemented with data from measurements taken at other locations. The method described above offers high measurement efficiency and is suitable for rapid underwater terrain surveying and reconstruction in irregular water bodies.

**Figure 17:**
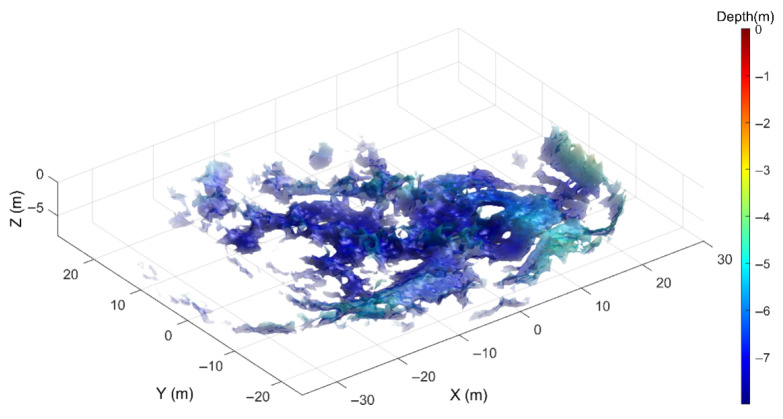
Underwater terrain rendering results.

Figure 17 shows a three-dimensional perspective of the merged survey data after image rendering. This presents an intuitive visualization of the underwater terrain, which greatly facilitates user observation.

### 4.4. Comparative Validation Tests

To validate the effectiveness of the proposed method, a comparative test using a single-beam bathymetric surveying system SDE-230 was conducted. Developed by South Surveying & Mapping (China), the SDE-230 represents a high-precision digital echo sounder engineered for rigorous hydrographic and geophysical applications. Operating at 200 kHz with a 5° beam width, it achieves a depth range of 0.3–600 m and a resolution of 1 cm, ensuring detailed seabed characterization. Its accuracy follows 0.01 m ± 0.1%D (where D denotes depth), supported by adjustable output power up to 300 W and a configurable sound velocity range (1300–2000 m/s).

**Figure 18:**
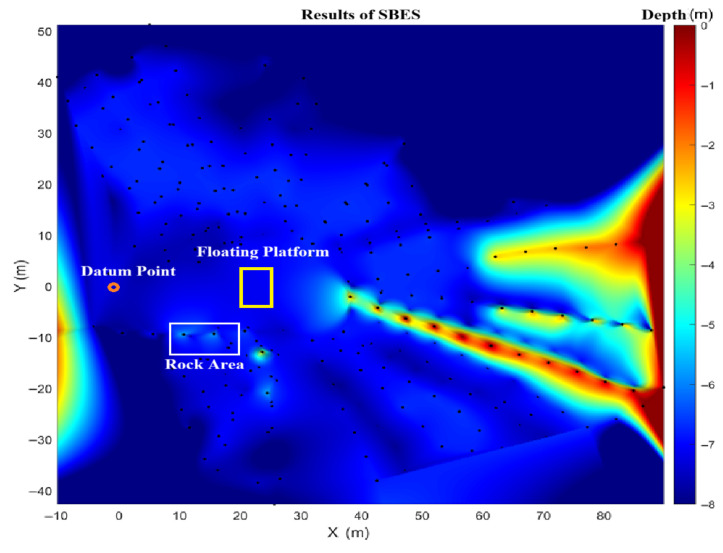
The bathymetric map generated through interpolation of point-by-point measurements from SDE-230. The black dots in the figure represent the actual measurement points, and the entire figure is formed by interpolating these measurement points.

As shown in Figure 18, the SDE-230 sonar indicates a maximum depth of approximately 8 m. The bathymetric sonar pole was equipped with a Differential GPS (DGPS) antenna at its apex. Affected by the lakeshore terrain, the GPS began providing stable positioning output only after the vessel moved a certain distance from the shore (refer to the Datum Point in Figure 18).

**Figure 19:**
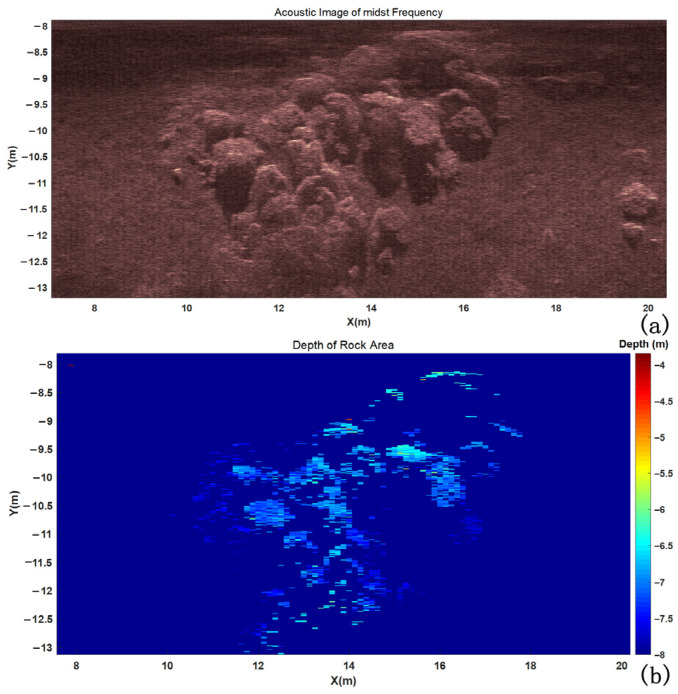
A comparison of measurement results from the two systems. (**a**) Sonar image scanned by the proposed system. (**b**) Measurement results by SDE-230.

The area within the horizontal coordinate range of 8 m to 20 m and the vertical coordinate range of −8 m to −13 m was chosen for comparative validation test. A detailed sonar scan image of the target area is generated, using the surveying methodology employing the unmanned surface vessel (USV) integrated with the XYT3000 sonar, as proposed in this study, and a comparative analysis was conducted with the scan image acquired by the SDE-230 system, as shown in Figure 19.

Figure 19 reveals an area of rocky terrain on the lakebed, with the bathymetric sonar indicating depths between 5.5 and 7 m below the water surface. Based on the correspondence between depth data and bottom features, the results from both systems demonstrate significant correlation.

## 5. Conclusions

This work aims to advance underwater sensing technology and its practical utility in marine science and industry. The study validated the three-dimensional reconstruction project of the lake next to Mingliyuan Farm in Nansha, Guangzhou. The unmanned ship surveying system proposed in this paper has three contributions:

(1) A rotary scanning surveying system based on unmanned ships is developed, RTK&IMU sensing information is integrated and fused to achieve high-precision underwater surveying in shallow waters.
(2) The influence of the navigation direction and scanning rotation direction of the rotating scanning sonar on the scanning line coverage was calculated through simulation, and the scanning coverage area was affected by platform shaking and seabed inclination.
(3) Through actual measurements at three stations at the bottom of the lake, the repeated elevation deviation of a certain point in the coverage area was less than 25 cm, verifying the practicality of this method in engineering.

This paper represents an application of a multibeam scanning sonar, originally designed for biomass assessment in marine ranching cages to underwater scene reconstruction. Through shallow-water experiments, this sonar can image not only fish schools but also fishnets and seafloor structures. Which expands the feasibility of this sonar for underwater surveying, structural sensing, and scene reconstruction. Achieving synergistic technological iteration between the sonar and a USV-based underwater detection system.

Quantitatively, the proposed system achieves range and angular resolutions that allows detection of individual moving targets and mesh structures, which is a capability not typical for conventional surveying sonars. Algorithmically, the framework integrates RTK/IMU fusion, beamforming, ENU/WGS84 transformations, and multi-view stitching in a lightweight manner tailored to this specific sonar’s characteristics.

The research group will continue to focus on three key areas in future works:

1. Developing advanced filtering algorithms for sonar point cloud data and implementing AI-based reconstruction techniques to enhance underwater imaging accuracy and resolution.
2. Conducting practical case studies to demonstrate the real-world deployment and effectiveness of our enhanced sonar processing methods in specific underwater scenarios.
3. Integrating the processed sonar data and analysis results into our existing Marine Geographic Information System (GIS) platform. This will involve expanding its spatial database capabilities and analytical tools to better support marine resource exploration and environmental monitoring.

## Figures and Tables

**Table 1:** XYT3000 sonar Specifications.

Indicator Item	Values
Operation Frequency	1 MHz
Range	≥30 m
Horizontal Opening Angle	360°
Vertical Opening Angle	≥65°(−5° to −70°)
Distance Resolution	≤1.0 cm
Horizontal Angular Resolution	≤0.5°@1 MHz
Vertical Angular Resolution	≤2.0°@1 MHz
Circular Scan Rate	≥4 RPM

**Table 2:** Steel Ball Position Estimated Results.

Slant Range (m)	Horizontal Bearing (°)	Vertical Depression Angle (°)
6.762	−0.255	31.702
6.762	−0.236	32.053
6.762	−0.255	32.068
6.762	−0.236	32.145
6.762	−0.236	32.133
6.762	−0.236	31.974
6.762	−0.236	32.2
6.762	−0.255	32.246
6.762	−0.255	32.136
6.762	−0.255	32.16
6.762	−0.255	32.122
6.762	−0.255	32.084
6.762	−0.255	32.114
6.762	−0.255	31.973
6.762	−0.255	32.095
6.762	−0.255	31.861
6.762	−0.255	31.995
6.762	−0.255	31.946
6.762	−0.255	31.96
6.762	−0.255	31.892
Average value: 6.762	−0.25	32.038
standard deviation: 0	0.008	0.129

**Table 3:** GNSS/RTK Receiver Specifications.

Parameter	Specification
Azimuth alignment accuracy	0.1°/2 m baseline (static, dual-antenna, RMS) or 0.2° (dynamic, with azimuth maneuvering, RMS)
Azimuth hold accuracy	2°/h
Horizontal attitude accuracy	0.3° (integrated navigation, RMS)
Integrated position accuracy	Horizontal: 1.5 m, vertical: 2.5 m (single-point satellite positioning, good signal conditions, RMS)
Integrated velocity accuracy	≤0.03 m/s
Angular rate range	±300°/s
Angular rate bias stability (10 s)	Azimuth axis: 1.5°/h, horizontal axis: 3°/h
Acceleration range	±8 g
Acceleration bias stability (10 s)	80 µg
Output update rate: Navigation data	200 Hz, IMU data: 2 kHz (customizable)

**Table 4:** Depth Comparison.

XY Coordinate (−0.85, −2.65)	Survey Point A	Survey Point B	Survey Point C
Depth	6.76 m	6.64 m	6.51 m

## Data Availability

The data presented in this study are available on request from the corresponding author.

## Abbreviations

The following abbreviations are used in this manuscript:

RTK	Real-Time Kinematic
IMU	Inertial Measurement Unit
USV	Unmanned Surface Vehicle
UUV	Unmanned Underwater Vehicle
DVL	Doppler Velocity Log
